# Deferoxamine Exhibits Antimicrobial and Immunomodulatory Activity Against *Mycobacterium abscessus*: Integrated In Silico and In Vitro Evidence

**DOI:** 10.3390/ijms27135789

**Published:** 2026-06-26

**Authors:** Roseane Lustosa de Santana Lira, Fabiane Barbosa Mendes, Pedro Lucas Brito Tromps Roxo, Joana Tenório Albuquerque Madruga Mesquita Meireles Teixeira, Caio César Santana de Azevedo, Arícia de Azevedo Vidigal, Eleonôra Costa Monteiro Gimenes, Reidson Stanley Soares dos Santos, Rivaldo Lira Filho, Camila Evangelista Carnib Nascimento, Flávia Danyelle Oliveira Nunes, Mayane Cristina Pereira Marques, José Lima Pereira-Filho, Carmem Duarte Lima Campos, Valério Monteiro-Neto, Rafael Cardoso Carvalho, Eduardo Martins de Sousa

**Affiliations:** 1 Postgraduate Program in Health Sciences, Federal University of Maranhão—UFMA, São Luís 65080-805, Brazil; roseane.lustosa@discente.ufma.br (R.L.d.S.L.); rivaldo.lira@ufma.br (R.L.F.); camila.carnib@ufma.br (C.E.C.N.); flavia.danyelle@ufma.br (F.D.O.N.); marques.mayanne@gmail.com (M.C.P.M.); jlp.filho@discente.ufma.br (J.L.P.-F.); carmem.campos@discente.ufma.br (C.D.L.C.); valerio.monteiro@ufma.br (V.M.-N.); carvalho.rafael@ufma.br (R.C.C.); 2 Postgraduate Program in Biosciences Applied to Health, CEUMA University—UNICEUMA, São Luís 65075-120, Brazil; fabianem807@gmail.com; 3 Medical School, CEUMA University—UNICEUMA, São Luís 65075-120, Brazil; pedrotromps.med@gmail.com (P.L.B.T.R.); tenoriojoana04@gmail.com (J.T.A.M.M.M.T.); caioazevedo1514@gmail.com (C.C.S.d.A.); aricia051769@ceuma.com.br (A.d.A.V.); eleonoragimenes.medicina@gmail.com (E.C.M.G.); 4 Postgraduate Program in Dentistry, CEUMA University—UNICEUMA, São Luís 65075-120, Brazil; reidsonstanley@gmail.com

**Keywords:** *Mycobacterium abscessus* subsp. *massiliense*, deferoxamine, iron chelation, antimicrobial resistance, adjuvant therapy

## Abstract

*Mycobacterium abscessus* subsp. *massiliense* (Mabs) is an emerging nontuberculous mycobacterium associated with difficult-to-treat infections due to intrinsic antimicrobial resistance, intracellular persistence, biofilm formation, and limited responsiveness to currently available therapeutic regimens. In this context, adjuvant strategies targeting iron-dependent metabolic pathways and metal homeostasis may enhance the efficacy of conventional antimicrobials. This study investigated deferoxamine (DFO), a clinically approved iron chelator, as a potential adjuvant against Mabs using integrated in vitro and in silico approaches. Cytocompatibility was assessed using an MTT assay in RAW 264.7 macrophages and a hemolysis assay in human erythrocytes. Antimicrobial activity was evaluated through minimum inhibitory concentration (MIC) and minimum bactericidal concentration (MBC) assays, while interactions with clarithromycin (CLA) and amikacin (AMK) were assessed using the checkerboard method. Effects on virulence-associated phenotypes were examined through biofilm formation assays and protein quantification in extracellular vesicle-enriched fractions. Intracellular activity and modulation of inflammatory mediator gene expression were investigated in Mabs-infected RAW 264.7 macrophages through colony-forming unit (CFU) recovery and reverse transcription quantitative polymerase chain reaction (qPCR). DFO exhibited low cytotoxicity and negligible hemolytic activity under the tested conditions. Direct antimicrobial testing revealed a predominantly bacteriostatic profile (MIC = 9.75 µg/mL; MBC > 10 mg/mL), whereas checkerboard analysis suggested a synergistic interaction with CLA (FICI = 0.047), which requires further confirmation by time-kill or CFU-based combination assays. Furthermore, DFO reduced biofilm biomass, decreased protein levels in vesicle-enriched fractions, lowered intracellular bacterial burden, and modulated cytokine gene expression in infected macrophages. Molecular docking, ADME/Tox, and PASS analyses generated exploratory hypotheses regarding potential molecular interactions and pharmacological properties. Overall, these findings support DFO as a promising experimental adjuvant candidate for further investigation against Mabs, particularly in combination with clarithromycin. However, confirmation of a putative iron-restriction-associated mechanism and its translational relevance will require validation in additional clinical isolates, iron-rescue experiments, mature biofilm models, and in vivo studies.

## 1. Introduction

Nontuberculous mycobacteria (NTM) comprise a broad and heterogeneous group of opportunistic microorganisms widely distributed in natural and built environments, including water, soil, moist surfaces, and healthcare-associated systems. Although NTM infections are classically associated with immunocompromised individuals, these microorganisms can also cause disease in susceptible immunocompetent hosts, particularly in the presence of structural lung disease, recurrent environmental exposure, or invasive procedures. Clinically, NTM are associated with chronic pulmonary disease, skin and soft tissue infections, osteoarticular involvement, and healthcare- or procedure-associated infections [1,2,3]. Within this group, the *Mycobacterium abscessus* complex (MABC) is of particular clinical concern because of its therapeutic complexity, especially in patients with cystic fibrosis, bronchiectasis, chronic pulmonary disease, immunosuppression, or a history of invasive procedures [1,2,3,4].

*Mycobacterium abscessus* subsp. *massiliense* (Mabs) has been associated with persistent respiratory, skin, and soft tissue infections, reflecting its ability to survive within macrophages, modulate host inflammatory responses, and form bacterial aggregates or biofilms that can impair antimicrobial penetration and reduce treatment efficacy. The virulence and persistence of *Mabs* are influenced by colony morphotype and surface lipid composition. Smooth variants contain abundant surface glycopeptidolipids (GPLs), which contribute to sliding motility, aggregation, biofilm formation, and immune evasion. In contrast, rough variants are typically associated with a loss or reduction in GPL expression, favoring cord formation, increased invasiveness, intracellular persistence, and exacerbated inflammatory responses [5,6].

Therapeutic management of Mabs infections is particularly complex because this pathogen combines intrinsic and acquired mechanisms of antimicrobial resistance, including low mycobacterial cell envelope permeability, efflux pump activity, drug-modifying enzymes, intracellular metabolic adaptation, and biofilm-associated tolerance. Moreover, inducible macrolide resistance mediated by erm genes, intersubspecies variability in antimicrobial susceptibility, and the toxicity of prolonged multidrug regimens contribute to the limited efficacy and poor clinical predictability of conventional treatment [7,8,9,10].

Additionally, inducible macrolide resistance mediated by erm genes, intersubspecies variability in antimicrobial susceptibility, and the toxicity of prolonged multidrug regimens contribute to the limited efficacy and poor clinical predictability of conventional treatment. Recent clinical evidence continues to highlight the limited antimicrobial susceptibility of Mabs and the therapeutic challenges associated with both pulmonary and extrapulmonary infections [11].

Recent evidence has strengthened the rationale for targeting iron acquisition and virulence-associated pathways in *Mabs*. Siderophore-mediated iron uptake, particularly pathways related to mycobactin biosynthesis and salicylate synthase activity, has been proposed as a promising strategy to limit *M. abscessus* virulence and metabolic fitness [12,13]. This approach is further supported by recent reviews highlighting iron uptake, ESX secretion systems, MmpL-associated transport, lipid remodeling, biofilm formation, and intracellular adaptation as relevant virulence-associated processes that may be exploited therapeutically [6]. In addition, gene-silencing approaches during infection have reinforced the importance of experimentally validating essential and infection-associated pathways in Mabs, supporting the use of target-directed strategies alongside phenotypic antimicrobial assays [14]. In this context, iron chelation by DFO may represent an adjuvant strategy that can impose metal-restriction stress on *Mabs*, potentially affecting iron-dependent metabolism, intracellular persistence, and susceptibility to conventional antibiotics.

Prior evidence from studies using iron chelators and iron metabolism-targeting compounds, such as deferiprone, gallium-protoporphyrin formulations, and VLX600, indicates that perturbing iron acquisition pathways may impair Mabs viability, even within intracellular infection models, and support the development of combination-based antimicrobial strategies [15,16,17]. Despite these findings, the specific activity of DFO against Mabs remains insufficiently characterized, particularly with respect to its interaction with clinically relevant antibiotics, its effects on virulence-associated phenotypes, and its ability to modulate the inflammatory response of infected host cells.

To address these gaps, we investigated DFO as a potential adjuvant agent against Mabs using integrated in vitro and in silico approaches. Specifically, we assessed its effects on bacterial growth, its interactions with amikacin (AMK) and clarithromycin (CLA), virulence-associated phenotypes, intracellular survival, and the inflammatory response of infected macrophages. We hypothesized that DFO-mediated iron restriction may disrupt iron-dependent bacterial processes and metal homeostasis, thereby impairing Mabs growth, biofilm formation, and intracellular persistence while increasing susceptibility to conventional antibiotics. Molecular docking was used exclusively as an exploratory approach to support mechanistic hypothesis generation and was not considered direct evidence of target inhibition.

## 2. Results

### 2.1. Biological Safety Profile in Host Cells

**Figure 1:**
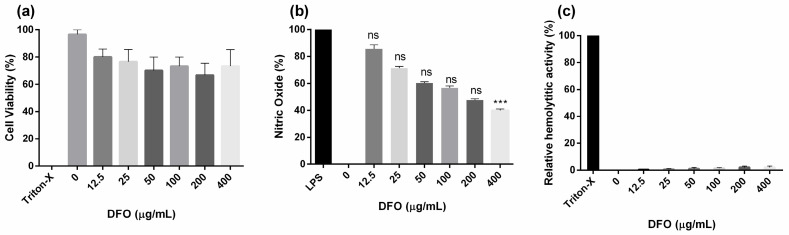
**Biological safety and immunomodulatory profile of DFO in host cell models.** (**a**) Viability of murine RAW 264.7 macrophages after 24 h of treatment with DFO, showing no statistically significant reduction compared with the untreated control (ns, *p* > 0.05). Triton X-100 was used as a positive cytotoxicity control. (**b**) Nitric oxide (NO) production, estimated by nitrite accumulation in the culture supernatant, in activated macrophages after treatment with DFO. LPS-stimulated cells were used as the reference control (100%) for NO production. (**c**) Relative hemolytic activity of human erythrocytes after exposure to DFO, showing values below the 2% threshold across all tested concentrations, consistent with negligible hemolytic activity. Data are presented as mean ± standard deviation (SD) from three independent biological experiments, each performed in technical triplicate. Statistical analysis was performed using a one-way ANOVA followed by Tukey’s post hoc test. *** *p* < 0.001 indicates a significant difference compared with the LPS-stimulated control; ns indicates *p* > 0.05 compared with the corresponding untreated controls.

DFO exhibited an initial cytocompatibility profile characterized by preserved RAW 264.7 macrophage viability and negligible hemolytic activity under the tested conditions. In RAW 264.7 macrophages exposed to DFO for 24 h, no statistically significant reduction in cell viability was observed compared with the untreated control (Figure 1a; *p* > 0.05). Cell viability remained high across the tested concentration range, with approximate values ranging between 68% and 80% from 12.5 to 400 µM. As expected, Triton X-100 markedly reduced cell viability, confirming the responsiveness of the assay.

Nitric oxide (NO) production, estimated by nitrite accumulation in culture supernatants, decreased after DFO treatment in activated macrophages (Figure 1b). The LPS-stimulated group was used as the reference condition and set at 100% relative NO production. DFO reduced NO levels in a concentration-dependent manner, with approximate values of 85% at 12.5 µM, 65% at 25 µM, 60% at 50 µM, 55% at 100 µM, 48% at 200 µM, and 40% at 400 µM. The most pronounced reduction was observed at 400 µM, which was statistically significant compared with the LPS-stimulated control (*p* < 0.001). These findings indicate that DFO reduced nitrite accumulation in activated macrophage cultures. However, the functional implications of this reduction for antimycobacterial host defense require further investigation.

In the hemolysis assay using human erythrocytes, DFO did not induce significant hemolytic activity at any of the tested concentrations (Figure 1c). Triton X-100 produced near-complete hemolysis, as expected for the positive control, whereas erythrocytes exposed to DFO maintained relative hemolytic activity close to baseline and below the 2% threshold across the concentration range. Together, these results indicate that DFO does not significantly impair RAW 264.7 macrophage viability, exhibits negligible hemolytic activity, and attenuates NO production in activated macrophages under the experimental conditions tested.

### 2.2. Direct Antimicrobial Activity

After establishing the initial biological safety profile of DFO, its direct antimicrobial activity against Mabs was evaluated. In vitro susceptibility testing revealed a minimum inhibitory concentration (MIC) of 9.75 µg/mL and a minimum bactericidal concentration (MBC) of >10 mg/mL for DFO (Table 1), resulting in an MBC/MIC ratio of >1025. This profile indicates predominantly bacteriostatic activity under the experimental conditions tested. In contrast, AMK and CLA exhibited MBC/MIC ratios of 1, consistent with bactericidal activity. Because DFO inhibited bacterial growth but did not exhibit bactericidal activity at the highest concentration tested, its use in combination with conventional antibiotics was further investigated as a potential adjuvant approach to enhance antimicrobial efficacy, rather than as a monotherapy.

### 2.3. Synergy with Antibiotics

**Figure 2:**
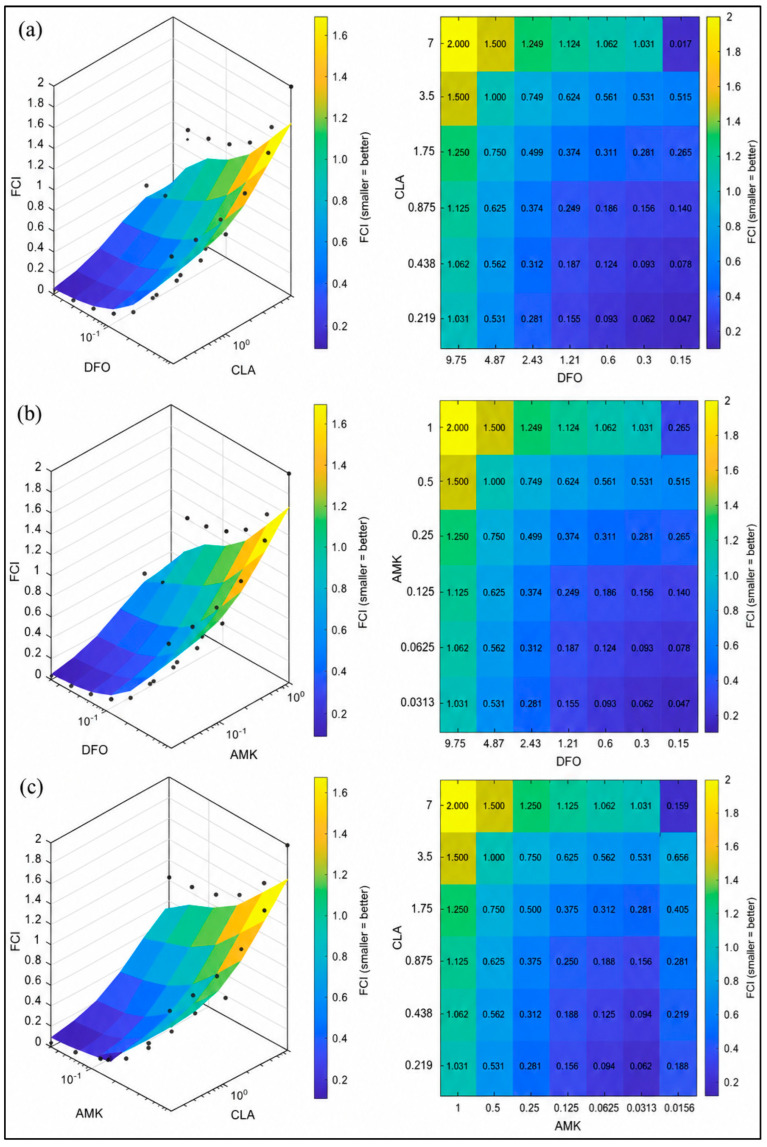
**Antimicrobial interactions against Mabs evaluated by the checkerboard assay.** (**a**) DFO + CLA; (**b**) DFO + AMK; (**c**) AMK + CLA. DFO, deferoxamine; AMK, amikacin; CLA, clarithromycin. Three-dimensional response surfaces and heat maps represent the fractional inhibitory concentration index (FICI) values calculated from the fractional concentrations of each compound in combination. Interaction patterns were interpreted according to the following criteria: synergy, FICI ≤ 0.5; additive effect, 0.5 < FICI ≤ 1.0; indifference, 1.0 < FICI ≤ 4.0; and antagonism, FICI > 4.0. Lower FICI values indicate stronger inhibitory interactions between the tested compounds. Data represent FICI values derived from three independent biological experiments performed in technical triplicate; mean FICI values and raw checkerboard matrices are provided in Supplementary Table S1 [19].

Because DFO displayed predominantly bacteriostatic activity as a single agent, we next evaluated whether its combination with conventional antibiotics could modulate the antimicrobial response against Mabs (Figure 2). Checkerboard assays revealed distinct interaction profiles among the tested combinations. The DFO + CLA combination yielded the lowest fractional inhibitory concentration index (FICI) value observed in the study (FICI = 0.047), suggesting a synergistic interaction under the checkerboard conditions used (Figure 2a). The DFO + AMK combination also shifted the FICI distribution toward lower values across parts of the concentration matrix, exhibiting values consistent with an additive interaction in the most active regions of the checkerboard (Figure 2b). In contrast, the AMK + CLA combination showed predominantly additive-to-indifferent interaction patterns, with higher FICI values across several concentration pairs (Figure 2c).

These results suggest that DFO may enhance the in vitro activity of selected antibiotics against Mabs, particularly CLA. However, given the exploratory nature of checkerboard assays and the remarkably low FICI observed for DFO + CLA, this interaction should be interpreted cautiously. This interpretation is consistent with recent evidence showing that drug–drug interactions in *M. abscessus* may be highly isolate-dependent, reinforcing the need to validate putative synergistic combinations across multiple strains and using complementary CFU-based or time-kill assays [18].

### 2.4. Effects on Biofilm Formation and Extracellular Vesicle-Enriched Fractions

**Figure 3:**
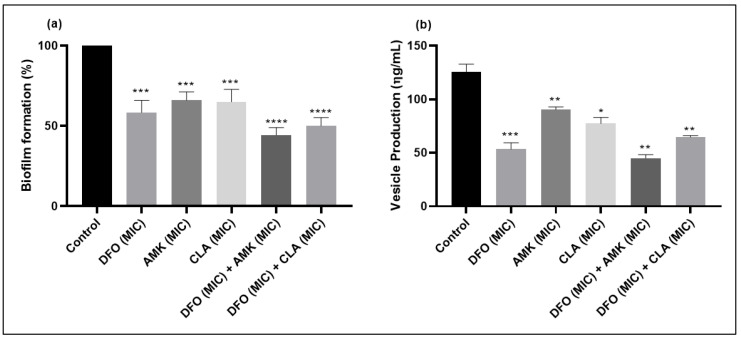
**Effects of DFO and antibiotic combinations on virulence-associated phenotypes of Mabs.** (**a**) Remaining biofilm biomass after treatment with DFO (MIC: 9.75 µg/mL), AMK (MIC: 1 µg/mL), CLA (MIC: 7 µg/mL), and their combinations. Values are expressed as percentage relative to the untreated control, set as 100%; lower values indicate reduced biofilm biomass under the experimental conditions tested. (**b**) Protein concentration associated with extracellular vesicle-enriched fractions obtained from cultures treated under the same conditions. Data are presented as mean ± standard deviation from three independent biological experiments, each performed in technical triplicate. Statistical analysis was performed using one-way ANOVA followed by Tukey’s post hoc test. * *p* < 0.05, ** *p* < 0.01, *** *p* < 0.001, and **** *p* < 0.0001 versus the untreated control.

DFO, alone or combined with antibiotics, reduced virulence-associated phenotypes of Mabs, including biofilm biomass (Figure 3). At the MIC, DFO reduced remaining biofilm biomass to approximately 58%, compared with 65% for AMK and 66% for CLA (Figure 3a). The combinations produced greater reductions, particularly DFO + AMK, with approximately 44% remaining biomass, followed by DFO + CLA, with approximately 50%.

Protein content in extracellular vesicle-enriched fractions was reduced after treatment (Figure 3b). Compared with the untreated control, which showed approximately 125 ng/mL, DFO reduced protein levels to approximately 52–55 ng/mL, while AMK and CLA reached approximately 90 ng/mL and 78 ng/mL, respectively. Among the combinations, DFO + AMK showed the lowest protein concentration, approximately 44 ng/mL, followed by DFO + CLA, with approximately 65 ng/mL.

These findings indicate reduced biofilm-associated biomass, as assessed by crystal violet staining, but do not demonstrate reduced viability of sessile bacilli or eradication of mature biofilms. Similarly, because vesicle number, size distribution, and morphology were not directly quantified, the reduced protein content should not be interpreted as direct evidence of decreased extracellular vesicle production.

### 2.5. Effect of DFO on the Intracellular Survival of Mabs in RAW 264.7 Macrophages

**Figure 4:**
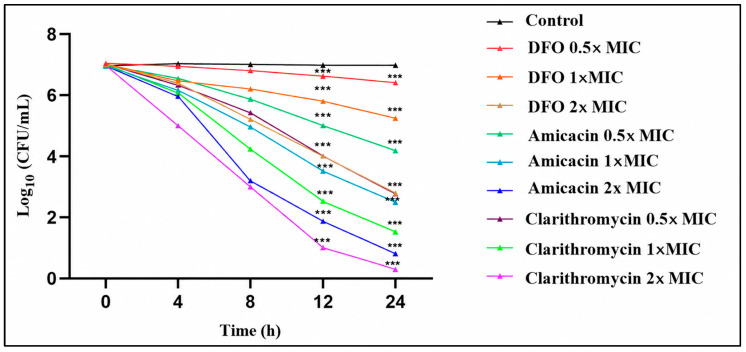
**Intracellular survival kinetics of Mabs in RAW 264.7 macrophages after treatment with deferoxamine, amikacin and clarithromycin at 0.5×, 1×, and 2× MIC.** The intracellular bacterial burden was quantified by CFU recovery at 0, 4, 8, 12, and 24 h after treatment and expressed as log_10_ CFU/mL. Data are presented as mean ± standard deviation (SD) from three independent biological experiments, each performed in technical triplicate. Statistical analysis was performed using a two-way ANOVA followed by Tukey’s post hoc test to assess the effects of treatment, time, and their interaction. *** *p* < 0.001 versus the untreated infected control at the corresponding time point.

The intracellular activity of DFO against Mabs was evaluated in infected RAW 264.7 macrophages over 24 h (Figure 4). Following infection, extracellular bacilli were minimized by serial phosphate-buffered saline (PBS) washes followed by gentamicin exposure, thereby minimizing the contribution of extracellular bacteria to colony-forming unit (CFU) recovery. In the untreated infected control, the bacterial burden remained nearly stable throughout the experimental period, with values close to 7.0 log_10_ CFU/mL from 0 to 24 h, confirming the intracellular persistence of Mabs.

DFO treatment reduced intracellular bacterial recovery in a concentration- and time-dependent manner. At 0.5× MIC, DFO produced only a modest reduction, with bacterial counts remaining close to 6.5 log_10_ CFU/mL after 24 h. At 1× MIC, an intermediate reduction was observed, reaching approximately 5.2 log_10_ CFU/mL at 24 h. The greatest inhibitory effect among the DFO-treated groups was observed at 2× MIC, with intracellular bacterial counts decreasing to approximately 2.7–2.9 log_10_ CFU/mL after 24 h, which was significantly lower than that of the untreated infected control.

The reference antibiotics produced greater reductions in intracellular CFU recovery than DFO at equivalent MIC multiples. AMK reduced intracellular bacterial counts to approximately 4.2 log_10_ CFU/mL at 0.5× MIC, 2.5–2.7 log_10_ CFU/mL at 1× MIC, and 0.8–1.0 log_10_ CFU/mL at 2× MIC after 24 h. CLA demonstrated the most pronounced reduction, reaching approximately 1.5 log_10_ CFU/mL at 1× MIC and falling below 0.5 log_10_ CFU/mL at 2× MIC. These findings indicate that DFO reduces the recovery of intracellular Mabs in RAW 264.7 macrophages under the tested conditions, although its effect was less pronounced than that of the reference antibiotics at higher concentrations. Because this assay does not directly establish the mechanism responsible for this reduction, additional studies, including iron-rescue assays and CFU-based combination assays, are required to determine whether iron restriction contributes to the observed intracellular effect.

### 2.6. DFO Modulates Cytokine Gene Expression in Mabs-Infected RAW 264.7 Macrophages

**Figure 5:**
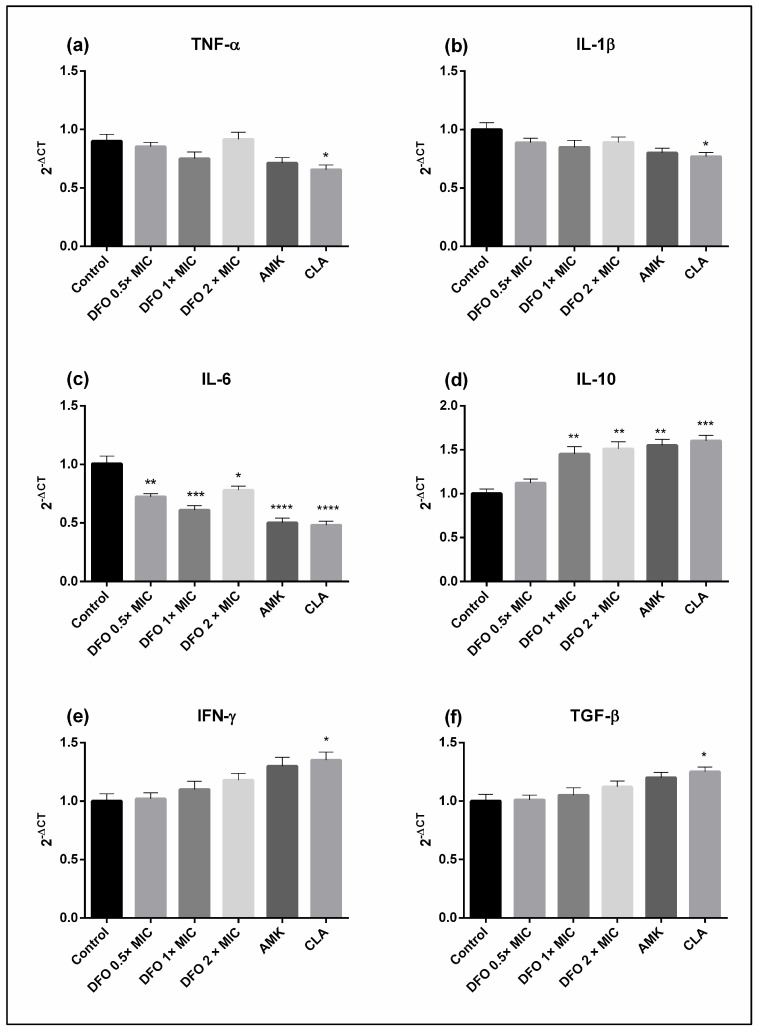
Relative expression of cytokine genes in *Mabs*-infected RAW 264.7 macrophages treated for 24 h with DFO at 0.5× MIC, 1× MIC, and 2× MIC, or with AMK and CLA. (**a**) TNF-α, (**b**) IL-1β, (**c**) IL-6, (**d**) IL-10, (**e**) IFN-γ, and (**f**) TGF-β. Data are presented as mean ± standard deviation (SD) from three independent biological experiments, each performed in technical triplicate. Statistical analysis was performed using a one-way ANOVA followed by Tukey’s post hoc test. * *p* < 0.01, ** *p* < 0.001, *** *p* < 0.0001, **** *p* < 0.00001 indicates a significant difference compared with the untreated infected control. These qPCR data reflect transcript abundance and do not directly indicate cytokine protein secretion.

FO treatment modulated cytokine-related gene expression in Mabs-infected RAW 264.7 macrophages (Figure 5a–f). Compared with the infected untreated control, DFO induced a concentration-dependent reduction in the relative expression of selected pro-inflammatory cytokine transcripts, although the magnitude of this effect varied among targets.

For TNF-α (Figure 5a), DFO treatment resulted in relative expression values of approximately 0.86-, 0.76-, and 0.93-fold at 0.5×, 1×, and 2× MIC, respectively, compared with the control group (set at 1.0). A significant reduction was observed only in the CLA-treated group (0.66-fold; *p* < 0.05), whereas AMK produced a moderate decrease (0.72-fold).

Similarly, IL-1β expression (Figure 5b) showed modest reductions following DFO treatment, with relative expression values of approximately 0.89-, 0.85-, and 0.90-fold at 0.5×, 1×, and 2× MIC, respectively. CLA significantly reduced IL-1β expression to 0.77-fold relative to the control (*p* < 0.05), while AMK reduced expression to approximately 0.80-fold.

The most pronounced effect of DFO was observed for IL-6 (Figure 5c). Relative expression decreased to approximately 0.73-fold (*p* < 0.01), 0.62-fold (*p* < 0.001), and 0.78-fold (*p* < 0.05) at 0.5×, 1×, and 2× MIC, respectively. AMK and CLA produced even greater reductions, reaching 0.50-fold and 0.48-fold, respectively (*p* < 0.0001).

In contrast, DFO increased the expression of regulatory cytokine-related transcripts. IL-10 expression (Figure 5d) increased to approximately 1.12-, 1.45-, and 1.52-fold at 0.5×, 1×, and 2× MIC, respectively, with significant increases observed at 1× and 2× MIC (*p* < 0.01). AMK and CLA also increased IL-10 expression to approximately 1.56-fold (*p* < 0.01) and 1.62-fold (*p* < 0.001), respectively.

Regarding IFN-γ (Figure 5e), DFO treatment produced a modest concentration-dependent increase, with relative expression values of approximately 1.02-, 1.10-, and 1.18-fold at 0.5×, 1×, and 2× MIC, respectively. The highest expression level was observed in the CLA-treated group (1.35-fold; *p* < 0.05), whereas AMK induced a moderate increase (1.30-fold).

A similar trend was observed for TGF-β (Figure 5f), with DFO-treated groups exhibiting relative expression values of approximately 1.01-, 1.05-, and 1.12-fold at 0.5×, 1×, and 2× MIC, respectively. CLA significantly increased TGF-β expression to 1.25-fold relative to the control (*p* < 0.05), whereas AMK induced a smaller increase (1.20-fold).

Collectively, these findings indicate that DFO modulates the transcriptional profiles of inflammatory and regulatory mediators in Mabs-infected macrophages, primarily by reducing IL-6 expression while promoting IL-10, and to a lesser extent IFN-γ and TGF-β, under the experimental conditions tested. Because these analyses were based exclusively on mRNA expression, the results should be interpreted as evidence of transcriptional modulation rather than direct changes in cytokine secretion or protein abundance. Further validation using protein-based approaches, such as ELISA, multiplex immunoassays, or flow cytometry, will be necessary to confirm DFO’s functional immunomodulatory effects.

### 2.7. Comparative Molecular Docking

Before interpreting the predicted DFO–target interactions, the docking protocol was validated by redocking co-crystallized ligands into representative binding sites. The heavy-atom root-mean-square deviation (RMSD) values obtained for Eis2/acetyl-CoA, InhA/NITD-916, and fumarase/malate were 0.707 Å, 1.055 Å, and 0.171 Å, respectively, which are all below the 2.0 Å threshold commonly used to indicate an acceptable reproduction of crystallographic binding poses. These results support the geometric reliability of the docking workflow, although they do not provide evidence of functional inhibition of the evaluated targets.

**Figure 6:**
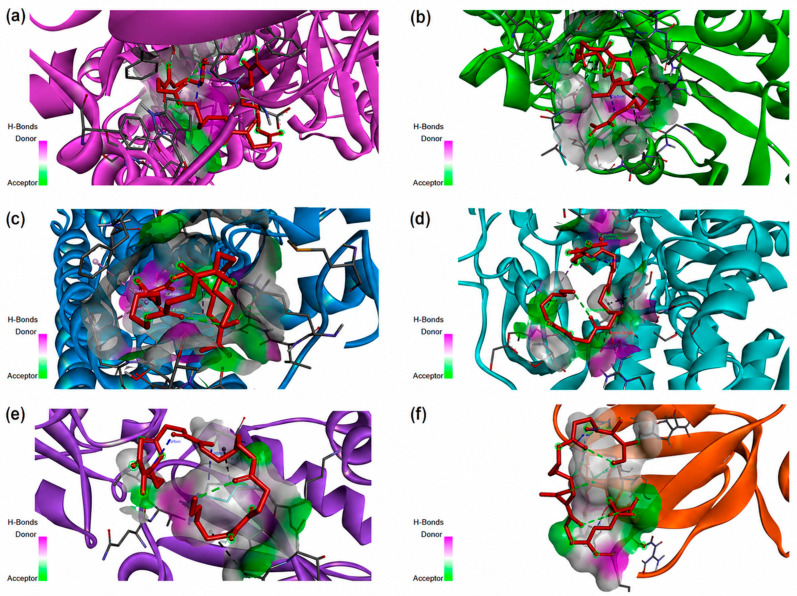
**Predicted docking interactions of DFO with selected Mabs proteins evaluated by molecular docking.** Molecular docking profiles within the binding sites of: (**a**) thioredoxin reductase (TrxB2; PDB ID: 4POB); (**b**) InhA (PDB ID: 7U0M); (**c**) fumarase (PDB ID: 3RRP); (**d**) enoyl-CoA hydratase/isomerase (PDB ID: 3R9Q); (**e**) Eis2 (PDB ID: 6RFT); and (**f**) the ε subunit of F-ATP synthase (PDB ID: 7XKZ). Free binding energies (ΔG) were calculated using AutoDock Vina 4. Note that these interactions represent structural in silico predictions rather than experimental validation of target inhibition.

To exploratorily evaluate the interaction potential of DFO, molecular docking was used to assess its predicted binding affinity toward molecular targets associated with Mabs survival, metabolism, and resistance. The analyses revealed more favorable predicted binding energies for the acetyltransferase Eis2 (−10.8 kcal/mol) and the enoyl-ACP reductase InhA (−10.3 kcal/mol), suggesting possible interactions with predicted or structurally defined binding sites (Figure 6). The predicted interactions involved mainly hydrogen bonds, hydrophobic contacts, and polar interactions between the hydroxamate/amine groups of DFO and binding site residues. However, in line with the exploratory nature of these analyses, these findings should not be interpreted as definitive proof of enzymatic inhibition; rather, they generate hypotheses that require subsequent experimental validation via enzyme activity assays, mutagenesis, biothermodynamics, and molecular dynamics simulations.

### 2.8. In Silico Exploratory Analyses and Predictions of Pharmacological and Biological Activity

The predicted biological activity spectrum, bioavailability, and pharmacokinetic profile of DFO were investigated to support mechanistic and translational interpretations. PASS analysis indicated a high probability of activity as an iron antagonist (Pa = 0.911; Table 2), which is consistent with its expected metal chelation mechanism. However, the insights derived from PASS, ADME/Tox, and molecular docking should be interpreted as predictive and complementary findings. While valuable for guiding hypotheses, these in silico results are not sufficient to replace experimental biochemical or pharmacological validation.

**Figure 7:**
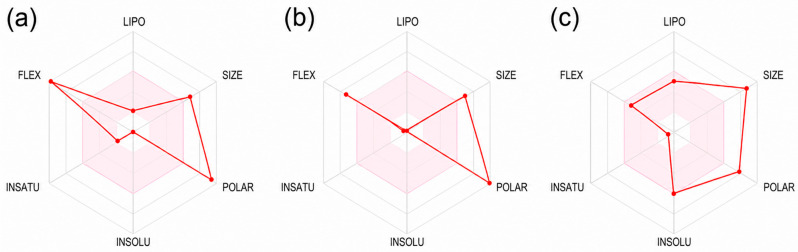
**Oral bioavailability radar plots.** (**a**) DFO; (**b**) AMK; and (**c**) CLA. The pink shaded area represents the optimal physicochemical space for oral bioavailability. The axes correspond to: lipophilicity (LIPO), molecular size (SIZE), polarity (POLAR), insolubility via log S scale (INSOLU), insaturation according to the fraction of sp3 carbons (INSATU), and flexibility based on the number of rotatable bonds (FLEX). Data were obtained from the SwissADME web server and should be interpreted as computational screening predictions.

As demonstrated in the bioavailability radar plot (Figure 7), DFO, similarly to AMK and CLA, exhibited an unfavorable predicted oral bioavailability profile (Table 3), mainly due to high polarity and structural flexibility. Also, Table 3 includes the main Lipinski-related descriptors, including molecular weight, hydrogen-bond donors, hydrogen-bond acceptors, lipophilicity, and the number of rule violations. DFO did not fully comply with Lipinski’s rule of five, primarily because of its high molecular weight, elevated hydrogen-bond donor count, and structural flexibility. These properties are consistent with poor predicted oral bioavailability and support the need for parenteral administration or targeted delivery strategies. This limitation does not preclude translational potential, as DFO is already clinically administered by parenteral routes. However, inhalable, liposomal, nanoparticulated, or phagosomal-targeted formulations should be explored before any clinical application in Mabs infections.

Furthermore, predicted nephrotoxicity should be interpreted with caution, particularly for deferoxamine, since clinically relevant nephrotoxicity has not been consistently reported under standard therapeutic use. In contrast, aminoglycosides such as AMK have well-established nephrotoxic potential, supporting the plausibility of the computational predictions.

## 3. Discussion

The present study investigated the antimicrobial and adjuvant potential of DFO against Mabs by integrating in vitro assays and in silico predictions. Overall, DFO showed low cytotoxicity and negligible hemolytic activity, a predominantly bacteriostatic profile, reduced biofilm biomass and protein content in extracellular vesicle-enriched fractions, decreased intracellular bacterial recovery in RAW 264.7 macrophages, and modulated cytokine-related gene expression. However, the results should be interpreted cautiously, as molecular docking, PASS, and ADME/Tox analyses do not establish enzymatic inhibition, target engagement, or a definitive mechanism of action.

The initial host–cell safety profile provides a basis for further investigation of DFO as a potential adjuvant candidate. Specifically, DFO did not significantly impair RAW 264.7 macrophage viability and maintained hemolysis below 2% across the evaluated concentration range, indicating negligible hemolytic activity according to standard hemocompatibility criteria [20]. Although this profile aligns with the established clinical use of DFO as an iron chelator for iron overload, its application in infectious settings requires specific evaluation of dosing, route of administration, local drug exposure, and safety in translational infection models.

DFO reduced nitrite accumulation in activated RAW 264.7 macrophages without marked cytotoxicity, suggesting that this effect was not primarily attributable to cytotoxicity under the tested conditions. Because DFO-mediated iron chelation may influence redox-sensitive pathways, macrophage activation, and intracellular iron-dependent signaling, this finding should not be interpreted as direct evidence of HIF-1α, iNOS, or intracellular iron modulation, which were not directly assessed. In mycobacterial infections, NO has context-dependent effects, contributing to antimicrobial host defense while also potentially promoting oxidative or nitrosative tissue injury when excessive [21]. Therefore, the functional relevance of this reduction requires further pathway- and protein-level validation.

A plausible explanation for the observed antimicrobial effects is that DFO restricts iron availability, thereby interfering with bacterial respiration, DNA synthesis, oxidative stress adaptation, biofilm formation, and intracellular persistence. This interpretation is consistent with the concept of nutritional immunity and with previous evidence supporting the use of iron chelators and metal-targeting compounds as antimicrobial adjuvants [22,23,24]. However, because intracellular iron availability, bacterial iron-regulated pathways, and iron-rescue conditions were not evaluated, the observed effects may reflect bacterial iron restriction, host–cell immunometabolic modulation, or both. Future studies should include iron supplementation, intracellular iron quantification, and targeted metabolic analyses to clarify the contribution of iron depletion to the DFO-associated phenotype.

The MIC observed for DFO confirms in vitro inhibitory activity against Mabs, but the high MBC indicates a predominantly bacteriostatic profile, rendering systemic monotherapy an unfeasible therapeutic strategy. Accordingly, DFO should be considered primarily as an adjuvant that limits bacterial growth, reduces persistence-associated phenotypes, and potentiates conventional antibiotics. This interpretation is reinforced by the limited oral bioavailability and short plasma half-life of DFO, whose clinical use relies primarily on parenteral administration [25,26]. For pulmonary or intracellular nontuberculous mycobacterial infections, targeted delivery strategies, such as inhaled formulations, liposomal systems, or macrophage-directed carriers, may be needed to enhance local exposure at the infection site. This rationale is supported by translational precedents involving inhaled liposomal AMK for NTM disease [27,28,29,30]. Pharmacokinetic and pharmacodynamic studies are therefore required to determine whether effective DFO concentrations can be achieved in lung tissue and phagosomal compartments.

Combination assays indicated the strongest interaction with DFO plus CLA, whereas DFO plus AMK showed a less pronounced but favorable interaction. These results suggest that DFO may enhance the in vitro activity of selected antibiotics against Mabs. Nevertheless, checkerboard assays provide only an initial pharmacodynamic signal and cannot define bactericidal kinetics, durability of effect, or mechanism. Time-kill studies, CFU-based combination assays, pharmacodynamic modeling, and in vivo infection models are required to confirm whether these interactions are reproducible and whether they result from iron-restriction-dependent changes in bacterial physiology, cell envelope homeostasis, energy metabolism, or intracellular stress responses [31,32].

The observed reduction in biofilm biomass, coupled with decreased protein levels in extracellular vesicle (EV)-enriched fractions, suggests that DFO may disrupt specific persistence-associated phenotypes in Mabs. However, because mature biofilms and EV metrics (number, size, or morphology) were not directly characterized, these findings must be interpreted strictly as evidence of reduced biofilm formation and altered protein cargo within vesicle-enriched preparations, rather than proof of biofilm eradication or direct inhibition of vesicle biogenesis [5,33]. This caution is further supported by recent evidence showing that host cell-derived EVs participate in Mabs–macrophage interactions and may regulate iron uptake in recipient macrophages during infection [34]. Future studies using mature biofilm models, sessile-cell viability assays, microscopy, nanoparticle tracking analysis, transmission electron microscopy, EV marker characterization, and proteomic or metal-content analyses are needed to define the antibiofilm and EV-related effects of DFO.

The reduction in intracellular bacterial recovery in RAW 264.7 macrophages supports the hypothesis that iron availability influences Mabs survival within the phagosomal environment. The removal of extracellular bacilli before treatment, together with preserved macrophage viability, reduces the likelihood that lower CFU recovery resulted solely from extracellular carryover or host–cell loss. However, DFO may also affect macrophage metabolism, intracellular iron redistribution, bacterial stress tolerance, or drug carryover during plating. Thus, the intracellular effect should be considered preliminary until confirmed using iron-rescue assays, human macrophages, host–cell viability monitoring during infection, and carryover controls.

DFO treatment also shifted cytokine-related transcriptional responses, with reduced TNF-α and IL-6 expression and increased IL-10 expression, suggesting a less pro-inflammatory profile. This interpretation is consistent with recent evidence indicating that the interaction between *Mabs* and immune cells is highly dynamic, involving macrophage activation, morphotype-dependent immune recognition, and context-dependent production of inflammatory mediators [35]. However, this response should not be automatically interpreted as beneficial. In mycobacterial infections, inflammatory mediators contribute to macrophage activation and pathogen control, whereas excessive suppression may impair host defense. Moreover, increased IL-10 expression warrants cautious interpretation, as recent findings indicate that Mabs can exploit IL-10-mediated pathways to inhibit autophagic flux via the MTOR/RUBCN axis and favor intracellular persistence [36]. Because the present analysis measured mRNA expression rather than secreted cytokines or autophagy-related markers, confirmation by ELISA, multiplex immunoassays, flow cytometry, autophagy assays, prolonged infection models, and in vivo experiments is required.

Lastly, in silico analyses suggested potential interactions between DFO and metabolic or resistance-associated targets, including Eis2, InhA, fumarase, and F-ATP synthase. Eis2 and InhA are biologically relevant because they are associated with aminoglycoside acetylation/resistance and mycolic acid biosynthesis/cell envelope maintenance, respectively. However, molecular docking has intrinsic limitations, as binding scores are computational estimates influenced by protein conformation, ligand preparation, docking parameters, scoring functions, and the inability to fully capture solvent effects, conformational flexibility, binding kinetics, metal coordination, or cellular complexity [37]. Therefore, the predicted DFO–Mabs protein interactions should be interpreted as hypothesis-generating findings. Although integrative approaches combining in vitro assays, molecular docking, and pharmacokinetic predictions are increasingly used in high-impact studies [38,39], these computational results provide only complementary mechanistic support and do not confirm direct enzymatic inhibition or functional target engagement.

Compared with other iron-targeting strategies, DFO has the advantage of an established clinical use, but it should be viewed within a broader group of compounds that interfere with iron metabolism in Mabs. Previous studies have shown that deferiprone combined with gallium-protoporphyrin, lipid-based delivery platforms, and VLX600 can reduce Mabs viability, including in intracellular infection models, supporting iron homeostasis and metal-dependent pathways as relevant adjuvant targets. However, comparisons among these approaches must consider differences in compound chemistry, iron-binding properties, cellular uptake, intracellular distribution, delivery systems, bacterial strain, infection model, and microenvironmental iron availability [15,16,17].

Overall, the convergence between in silico predictions and in vitro findings supports further evaluation of DFO in combination-based approaches against Mabs. DFO limited bacterial growth, reduced selected persistence-associated phenotypes, and showed low cytotoxicity. However, whether these effects result from bacterial iron restriction, host–cell modulation, or both remains to be determined.

This study has important limitations that warrant acknowledgment. First, all experiments were performed using a single Mabs strain displaying a smooth colony morphotype, which precludes immediate generalizability of the findings to other clinical isolates, rough variants, or additional members of the Mabs complex. Second, iron-rescue assays, experiments targeting pre-established mature biofilms, time-kill synergy studies, and experimental validation of the predicted Eis2 and InhA interactions were not conducted. Furthermore, the study did not incorporate human-derived macrophages or in vivo animal infection models. Consequently, the effects observed in RAW 264.7 macrophages must be interpreted as a preliminary in vitro indication rather than definitive evidence of translational efficacy. Finally, ADME/Tox predictions and molecular docking analyses remain strictly exploratory and should be regarded as hypothesis-generating support rather than causal evidence of target engagement or a definitive mechanism of action.

Future studies should evaluate DFO across an expanded panel of clinical isolates, encompassing both smooth and rough morphotypes, and incorporate iron supplementation or iron-rescue conditions to definitively determine whether the observed phenotypes are driven by iron restriction. Time-kill kinetics are necessary to validate the interactions of DFO with CLA and AMK, while mature biofilm models should be employed to ascertain whether DFO exerts activity against pre-established, sessile bacterial communities. Experimental validation of the predicted molecular targets should involve enzymatic, biophysical, or direct target-engagement assays. Additionally, translational applicability of DFO as an adjuvant candidate against Mabs will require evaluation in pulmonary infection models, cystic fibrosis-relevant physiological systems, human macrophage assays, and specialized drug delivery strategies designed to optimize intracellular and lung tissue exposure.

## 4. Materials and Methods

### 4.1. Microorganism and Culture Conditions

The Mabs clinical isolate BCCIPTSP Go01, a smooth-morphotype strain originally recovered from surgical site infections in Goiânia, Brazil, was used in all in vitro assays. The isolate was recovered from cutaneous abscess exudates of patients treated at private hospitals and subsequently identified as Mabs. Phenotypically, the strain exhibits a smooth morphotype, a feature critical for interpreting the assays, as this morphotype is associated with surface glycopeptidolipids, a propensity for aggregation, biofilm formation capabilities, and specific modulation of mycobacterium–host interactions.

The strain was preserved at −80 °C in Middlebrook 7H9 broth (Thermo Fisher Scientific™, Santa Fe, KS, USA) supplemented with sterile glycerol and reactivated on Middlebrook 7H11 agar (Thermo Scientific™, Santa Fe, KS, USA) supplemented with 10% oleic acid-albumin-dextrose-catalase (OADC) at 37 °C until isolated colonies were obtained.

For assay preparation, single colonies were inoculated into Middlebrook 7H9 broth (Thermo Scientific™, Santa Fe, KS, USA) supplemented with 10% OADC, 0.05% Tween 80, and glycerol, when applicable, and incubated at 37 °C with agitation until the exponential growth phase. Cultures were homogenized by vortexing and repeated passage through a sterile fine-gauge needle to minimize clumping. Suspensions were adjusted to OD625 = 0.1 and, when applicable, confirmed by CFU/mL quantification. Viability and purity were verified by plating on solid medium and monitoring colony morphology.

The utilization of this clinical isolate was authorized through a signed Informed Consent Form, in compliance with the protocol approved by the Institutional Research Ethics Committee (CAAE No. 21357413.4.0000.5084).

### 4.2. Selection of Compounds for Computational Analyses

The chemical structures of DFO (CID: 2973) (CRISTÁLIA, Itapira, SP, Brazil), AMK (CID: 377768) (Sigma-Aldrich^®^, St. Louis, MO, USA), and CLA (CID: 84029) (Sigma-Aldrich^®^, St. Louis, MO, USA) were obtained from the PubChem database (National Center for Biotechnology Information). These structures were utilized for all in silico analyses.

### 4.3. Preparation of the Bacterial Inoculum

An isolated Mabs colony was suspended in PBS containing 0.05% Tween 80 in tubes with sterile glass beads and homogenized by vortexing until complete dispersion was achieved. Turbidity was visually adjusted to the 0.5 McFarland scale (~1 × 10^8^ CFU/mL) and confirmed by spectrophotometric reading at 625 nm (Optical Density = 0.105). After resting for 30 min, 50 µL of the suspension was diluted in 9.95 mL of Mueller–Hinton broth (Merck, Darmstadt, Germany) to obtain a final concentration of 1 × 10^4^ CFU/mL [40].

### 4.4. Cytotoxicity and Cell Viability Assay in Raw 264.7 Murine Macrophages

DFO cytotoxicity was evaluated in the RAW 264.7 murine macrophage lineage using the MTT [3-(4,5-dimethylthiazol-2-yl)-2,5-diphenyltetrazolium bromide] method. Cells (1 × 10^5^ cells/well) were seeded in 96-well plates, cultured in DMEM (Dulbecco’s Modified Eagle Medium) (Sigma-Aldrich^®^, St. Louis, MO, USA) supplemented with 10% fetal bovine serum, and treated with varying concentrations of DFO (12.5–400 µM). Incubation was performed for 24 h at 37 °C in a 5% CO_2_ atmosphere without agitation. Culture medium alone served as the negative control, while 0.05% Triton X-100 was used as the positive control. Cell viability was quantified by measuring absorbance at 540 nm [40]. All experiments were performed in technical triplicate for each of the three independent biological replicates.

### 4.5. Determination of Hemolytic Activity

The hemocompatibility of DFO was evaluated using human erythrocytes obtained from packed red blood cell concentrates provided by the Hemocentro do Maranhão blood center, with approval from the CEUMA University Research Ethics Committee (CAAE No. 52245415.6.0000.5084). Erythrocytes were washed three times with PBS (pH 7.4), resuspended to 2% (*v*/*v*), and incubated with DFO (12.5–400 μM) for 60 min at 37 °C.

Erythrocytes incubated in PBS and in 0.05% Triton X-100 were used as negative and positive controls, respectively. After incubation, samples were centrifuged and the supernatant absorbance was measured at 540 nm to quantify hemoglobin release.

Hemolytic activity was categorized as non-hemolytic (<2%), slightly hemolytic (2–5%), or hemolytic (>5%) [20] and expressed relative to the Triton X-100 positive control and calculated using the following formula [40]:

$$\%\;hemolysis=\left[\frac{\left(As-Ab\right)\times \;100}{Ac-Ab}\right]$$

where Ab is the absorbance of the blank/negative control, As is the absorbance of the treated sample, and Ac is the absorbance of the Triton X-100 positive control.

All experiments were performed in technical triplicate for each of the three independent biological replicates.

### 4.6. Quantification of Nitric Oxide Production

Nitric oxide (NO) production was indirectly estimated by nitrite accumulation in culture supernatants using the Griess reagent assay. Murine RAW 264.7 macrophages were infected with Mabs at a multiplicity of infection (MOI) 10:1, stimulated with LPS (1 μg/mL), and treated with DFO (12.5–400 μM). After incubation, supernatants were clarified when necessary, mixed with Griess reagent, and read at 540 nm. Nitrite levels were calculated using a sodium nitrite standard curve or expressed relative to the infected/stimulated control.

Cells infected and stimulated with LPS but left untreated with DFO served as the positive control for NO production, whereas uninfected and unstimulated cells were utilized as the basal control [24].

All experiments were performed in technical triplicate for each of the three independent biological replicates.

### 4.7. Determination of MIC and MBC

The antimicrobial activities of DFO, AMK, and CLA against Mabs were evaluated by broth microdilution in sterile 96-well plates using Mueller–Hinton (Himedia, Mumbai, India) broth, adapted from susceptibility testing protocols for rapidly growing mycobacteria. Serial dilutions were prepared at the following concentration ranges: DFO, 10,000–4.87 μg/mL; AMK, 128–0.06 μg/mL; and CLA, 32–0.015 μg/mL.

DFO and AMK stock solutions were prepared in sterile ultrapure water. CLA was initially solubilized in dimethyl sulfoxide (DMSO) (Sigma-Aldrich^®^, St. Louis, MO, USA) and diluted in culture medium, with a final DMSO concentration ≤1% (*v*/*v*). Each well contained 200 μL, composed of 100 μL of compound dilution and 100 μL of standardized bacterial suspension, yielding a final inoculum of approximately 1 × 10^4^ CFU/mL. Growth, sterility, solvent, and inoculum-free compound controls were included.

Microplates were incubated at 37 °C for up to 5 days. Then, 0.01% resazurin was added and plates were incubated for an additional 24 h. Wells that remained blue/purple were interpreted as having no detectable metabolic activity, whereas a color shift to pink indicated viable growth. The minimum inhibitory concentration (MIC) was defined as the lowest compound concentration capable of preventing the color shift.

To ensure high reproducibility of the minimum bactericidal concentration (MBC), 10-μL aliquots from wells without detectable growth were subcultured on Middlebrook 7H11 agar (Thermo Fisher Scientific™, Santa Fe, KS, USA) supplemented with OADC and incubated at 37 °C for up to 5.

The reading timepoints were strictly differentiated between inoculum standardization, which utilized cultures grown for approximately 3 days, and the incubation of the MIC/MBC assays, which was maintained for up to 5 days depending on the observed mycobacterial growth.

Where applicable, the reduction in bacterial load was expressed as log_10_ CFU/mL. The logarithmic reduction was calculated as the difference between the initial bacterial load and the load recovered post-treatment: log_10_ reduction = log_10_ CFU/mL initial −log10 CFU/mL final. The percentage of bacterial death was calculated using the following equation [41]:

$$\%death=\frac{{CFU}_{initial}-{CFU}_{final}}{{CFU}_{initial}}\;\times 100$$

MBC was defined as the lowest concentration preventing visible colony growth, corresponding to ≥99.9% reduction in viable bacteria. Activity was classified as predominantly bactericidal when MBC/MIC ≤ 4 and predominantly bacteriostatic when MBC/MIC > 4 [42,43].

### 4.8. Synergy Evaluation (Checkerboard Method)

Antimicrobial interactions among DFO, AMK, and CLA were evaluated by the broth microdilution checkerboard method in sterile 96-well plates using Middlebrook 7H9 broth (Thermo Fisher Scientific™, Santa Fe, KS, USA) supplemented with 10% OADC and 0.05% Tween 80, adapted from standard antimicrobial susceptibility testing protocols for rapidly growing mycobacteria.

Bacterial suspensions were prepared from exponential-growth-phase cultures and adjusted to a final inoculum of approximately 1 × 10^5^ CFU/mL. Combinations were tested in 8 × 8 matrices with 200 μL per well, using concentrations based on the individual MICs: DFO, 2.44–39.0 µg/mL; AMK, 0.25–4.0 µg/mL; and CLA, 1.75–28.0 µg/mL.

The combinations evaluated were DFO + CLA, DFO + AMK, and AMK + CLA. Each plate included untreated growth controls, sterility controls, solvent controls where applicable, and wells containing the individual drugs. Plates were incubated at 37 °C for 3–5 days, and bacterial viability was assessed by visual inspection and 0.01% resazurin (Thermo Fisher Scientific™, Santa Fe, KS, USA), as described for MIC determination. The absence of a color shift (maintaining the blue/purple color) was interpreted as an absence of detectable bacterial growth, whereas a shift to pink indicated viable bacterial metabolism.

The MIC combination was defined as the lowest concentration that inhibited visible growth. For each antimicrobial pair, the Fractional Inhibitory Concentration Index (FICI) was calculated using the following equation [19,44,45]:

$$FICI=\frac{A}{{MIC}_{A}}\;+\frac{B}{{MIC}_{B}}$$

where A and B represent the concentrations of the compounds in the combination that resulted in the inhibition of bacterial growth, while MIC_A_ and MIC_B_ correspond to the MICs of the compounds tested individually.

The interpretation was based on the following criteria: FICI ≤ 0.5, synergism; 0.5 < FICI ≤ 1.0, additive effect; 1.0 < FICI ≤ 4.0, indifference; and FICI > 4.0, antagonism [19].

All assays were performed in independent triplicates, and final values were calculated from the raw checkerboard matrices (Supplementary Table S1).

### 4.9. Biofilm Formation and Extracellular Vesicle Production

The capacity of DFO, AMK, CLA, and their combinations to interfere with Mabs biofilm formation, was evaluated in 96-well, flat-bottom polystyrene microplates. For this assay, bacterial suspensions were prepared in Middlebrook 7H9 broth supplemented with 10% OADC without Tween 80, thereby promoting bacterial adhesion and subsequent biofilm development. The initial suspension was adjusted to approximately 1 × 10^7^ CFU/mL, and 200-μL aliquots were distributed into each well.

Treatments with DFO, AMK, CLA, or their respective combinations were added at the onset of the assay, immediately following bacterial inoculation, at concentrations determined from previously established MIC values. Untreated bacterial cultures served as biofilm formation controls, whereas wells containing sterile medium exclusively were used as sterility controls. Solvent controls were included at the same final concentrations used in the treatment groups, where applicable. The plates were incubated statically at 37 °C in a humidified chamber for 5 days to allow for adhesion and biofilm maturation.

After incubation, wells were washed with PBS, stained with 0.1% crystal violet for 15 min, washed again, air-dried, and solubilized with 95% ethanol. Absorbance was read at 570 nm, and results were expressed as the percentage of remaining biofilm biomass relative to the untreated control. This assay was interpreted as inhibition of biofilm formation rather than eradication of mature biofilms.

Extracellular vesicle (EV)-enriched fractions from Mabs cultures were obtained from bacterial cultures grown in Middlebrook 7H9 broth supplemented with 10% OADC under aerobic conditions at 37 °C. Standardized bacterial suspensions were inoculated into liquid medium and grown to the exponential phase, after which cultures were treated with DFO, AMK, CLA, or their MIC-derived combinations. Untreated cultures served as controls.

After incubation, cultures were centrifuged at 3800× *g* for 15 min at 4 °C to remove bacterial cells, aggregates, and large debris. The supernatants were then filtered through sterile 0.22-μm membranes and subjected to ultracentrifugation at 100,000× *g* for 1 h at 4 °C. The resulting EV-enriched pellets were washed with ice-cold sterile PBS and ultracentrifuged again under the same conditions to reduce soluble protein and medium contaminants. Final pellets were resuspended in sterile PBS and stored at −80 °C until analysis.

EV-associated protein content was indirectly quantified using the bicinchoninic acid (BCA) assay (Thermo Fisher Scientific™, Santa Fe, KS, USA), with absorbance measured at 562 nm. Results were expressed as protein concentration in the EV-enriched fraction and normalized to the bacterial load of the original culture, determined by CFU/mL counts.

All experiments were performed in three independent biological replicates, each conducted in technical triplicate.

### 4.10. Infection and Intracellular Survival in Murine RAW 264.7 Macrophages

For the intracellular infection model, RAW 264.7 (ATCC^®^ TIB-71) macrophages were seeded at 2 × 10^5^ cells/well in 24-well plates and incubated for 18–24 h to allow adhesion. Cells were infected with Mabs at an MOI of 10:1 (bacteria: macrophage) for 4 h at 37 °C and 5% CO_2_ [44,45,46].

After infection, monolayers were washed three times with sterile PBS and incubated with gentamicin (50 μg/mL) for 1 h to reduce extracellular bacilli. After additional washing, infected macrophages were treated with DFO, AMK, or CLA at 0.5×, 1×, and 2× MIC. Gentamicin-exposed infected controls were maintained to verify that the clearance step did not compromise intracellular CFU recovery.

Intracellular bacterial burden was determined at 0, 4, 8, 12, and 24 h post-treatment. Macrophages were lysed with 0.1% Triton X-100, serially diluted in PBS, and plated on Middlebrook 7H10/7H11 agar supplemented with OADC. Plates were incubated at 37 °C for 3–5 days, and results were expressed as log_10_ CFU/mL. Antimicrobial activity was interpreted according to log_10_ CFU/mL reduction relative to t = 0 h [2,40].

For intracellular bacilli recovery, infected macrophages were lysed with 500 μL of 0.1% Triton X-100 per well for 10 min at room temperature, followed by gentle homogenization. Gentamicin was used only after infection to eliminate residual extracellular bacteria, and infected controls exposed to the same gentamicin protocol were included to confirm that this step did not affect macrophage viability or intracellular bacilli recovery.

Bacterial load was determined by serial dilution of the lysates from 10^−1^ to 10^−8^ in PBS. Aliquots of 100 μL from each dilution were plated on Middlebrook 7H10 or 7H11 agar supplemented with OADC and incubated at 37 °C with 5% CO_2_ for 3–5 days. Plates containing 30–300 colonies were used for CFU counting. Log_10_ reduction was calculated using the following formula [46]:

$${Log}^{10}reduction={log}_{10}\left({CFU}_{t=0}\right)-\;{log}_{10}{CFU}_{t=24h}$$

Antimicrobial activity was interpreted according to the reduction relative to the initial inoculum. Based on international CLSI/EUCAST guidelines criteria, bactericidal activity was defined as a ≥3 log_10_ CFU/mL reduction, corresponding to 99.9% bacterial killing. Reductions of 1–3 log_10_ were classified as intermediate activity, whereas reductions <1 log_10_ indicated a bacteriostatic profile [46].

All experiments were performed in technical triplicate for each of the three independent biological replicates.

### 4.11. Cytokine Gene Expression Analysis

RAW 264.7 macrophages (ATCC^®^ TIB-71) were cultured in RPMI-1640 medium supplemented with 10% fetal bovine serum and 1% penicillin/streptomycin, and maintained at 37 °C in a humidified 5% CO_2_ atmosphere. Cells were infected with Mabs at a multiplicity of infection (MOI) of 10:1 and treated for 24 h with DFO at 0.5×, 1×, and 2× MIC (4.875–19.5 μg/mL), AMK (1 μg/mL), or CLA (0.007 μg/mL). This MOI was selected to ensure a homogeneous macrophage response to infection [47].

Total RNA was extracted using the RNeasy Mini Kit (Qiagen, Hilden, Germany) and quantified with a NanoDrop 2000 spectrophotometer (Thermo Fisher Scientific, Waltham, MA, USA). RNA purity was assessed using A260/A280 and A260/A230 ratios, and only samples within the 1.8–2.2 range were used for downstream analyses. Residual genomic DNA was removed with DNase I (Invitrogen, Carlsbad, CA, USA), and first-strand cDNA was synthesized using the High-Capacity cDNA Reverse Transcription Kit (Applied Biosystems, Foster City, CA, USA) [47,48].

qPCR was performed using SYBR Green PCR Master Mix on the QuantStudio 3 Real-Time PCR System to quantify TNF-α, IL-1β, IL-6, IL-10, IFN-γ, and TGF-β. Primer sequences are provided in Supplementary Table S2 [49]. Amplification efficiencies for all primer pairs were determined via standard calibration curves, ranging between 95% and 105%.

The β-actin gene was selected as the endogenous reference gene due to its previously validated expression stability in the RAW 264.7 cell line under mycobacterial infection conditions. Relative gene expression levels were calculated using the comparative 2^−ΔΔCt^ method. For this calculation, the infected but untreated macrophage group served as the calibrator (reference unit = 1.0), enabling the quantification of the impacts of DFO, AMK, and CLA on gene modulation [47,48].

Each qPCR reaction was performed in a final volume of 20 μL using SYBR Green PCR Master Mix, primers at 0.4 μM, cDNA template, and nuclease-free water. The cycling conditions included 95 °C for 10 min, followed by 40 cycles of 95 °C for 15 s and 60 °C for 60 s, with melting curve analysis from 60 °C to 95 °C to confirm amplicon specificity. No-template and no-reverse transcriptase controls were included for each primer pair. β-actin was used as the reference gene after confirming stable Ct variation below 1 cycle across groups and amplification efficiency between 95% and 105%.

Differences between groups were analyzed by one-way ANOVA followed by Tukey’s post hoc test, with statistical significance defined as *p* < 0.05.

All experiments were performed in technical triplicate across three independent biological replicates (*n* = 3).

### 4.12. Molecular Docking

The interaction potential between DFO, AMK, and CLA against Mabs protein targets was evaluated via molecular docking.

The chemical structures of the ligands were retrieved from the PubChem database (DFO CID: 2973; AMK CID: 37768; CLA CID: 84029) and prepared using Open Babel, which involved 3D conformational optimization via the MMFF94 force field and subsequent conversion to the PDBQT format [50].

Ligand structures were retrieved from PubChem (DFO CID: 2973; AMK CID: 37768; CLA CID: 84029) and prepared with Open Babel (https://openbabel.org/) (accessed on 15 December 2025) using MMFF94 3D optimization and PDBQT conversion [50]. Protein structures were retrieved from the RCSB Protein Data Bank (https://www.rcsb.org/), and the PDB IDs and direct links are provided in Table 4. Macromolecular targets were selected based on their relevance to energy metabolism, cell-wall biosynthesis, redox homeostasis, intracellular survival, and antimicrobial resistance, encompassing Eis2, InhA, TrxB2, fumarase, enoyl-CoA hydratase/isomerase, and the ε subunit of F-ATP synthase. Binding-site prediction was supported by PrankWeb (coordinates: x = 29.5, y = 7.2, z = 59.7) [51] (https://prankweb.cz/) (accessed on 15 December 2025), and docking simulations were performed with AutoDock Vina4 (https://vina.scripps.edu/) (accessed on 15 December 2025).

Ligand–receptor affinities were estimated from predicted free binding energy (ΔG), with lower values indicating more favorable computational interactions. Post-docking interactions were analyzed with BIOVIA Discovery Studio Visualizer 4.2 (https://www.3ds.com/products/biovia) (accessed on 20 December 2025) to identify key residues and interaction types, including hydrogen bonds, hydrophobic contacts, and polar interactions [50,52].

Validation of the docking protocol was conducted via re-docking assays utilizing co-crystallized ligands from the Mabs target proteins. Native ligands were extracted from their respective crystallographic complexes and subsequently re-docked into the identical binding sites originally described in the PDB deposited structures. These simulations were carried out under the exact methodological parameters employed in the primary docking experiments. The grid box was centered on the Cartesian coordinates of the co-crystallized ligand within the relevant catalytic or active center of each protein. Protocol validation was determined by calculating the heavy-atom root-mean-square deviation (RMSD) between the experimental (native) and predicted poses using BIOVIA Discovery Studio Visualizer 4.2. RMSD values below 2.0 Å were considered indicative of a successfully validated docking protocol [53].

### 4.13. ADME Analysis and Prediction of Toxicological Properties (In Silico)

Absorption, distribution, metabolism, and excretion (ADME) properties were evaluated using SwissADME (http://www.swissadme.ch/; accessed 30 April 2024) [54]. Molecular descriptors, including molecular weight, hydrogen-bond acceptors and donors, lipophilicity, molar refractivity, rotatable bonds, and topological polar surface area (TPSA), were analyzed using Lipinski-related criteria to estimate drug-likeness.

Toxicity profiles were predicted using admetSAR 2.0 (https://lmmd.ecust.edu.cn/admetsar2; accessed 14 April 2026) and ProTox 3.0 (https://tox.charite.de/protox3/; accessed 14 April 2026) [55,56]. Predicted toxicity endpoints, including nephrotoxicity, were interpreted strictly as preliminary computational screening results rather than clinically confirmed toxic effects.

### 4.14. Prediction of Biological Activities (In Silico)

Biological activity spectra were predicted using PASS Online (https://way2drug.com/PassOnline/; accessed 30 April 2024). Compound structures were retrieved from the PubChem database in SMILES (Simplified Molecular Input Line Entry System) format, and activities were considered relevant when the probability of activity (Pa) exceeded the probability of inactivity (Pi). Predictions were interpreted as high (Pa > 0.7), intermediate (0.5 ≤ Pa ≤ 0.7), or low (Pa < 0.5) probability of experimental confirmation [40,57,58].

### 4.15. Statistical Analysis

Data are presented as mean ± standard deviation (SD) from three independent biological experiments (*n* = 3), each performed in technical triplicate. Normality and homogeneity of variances were assessed prior to the selection of statistical tests, when applicable. Comparisons among multiple groups were performed using one-way or two-way analysis of variance (ANOVA), according to the experimental design and the number of factors evaluated, followed by Tukey’s post hoc test for multiple comparisons. Differences were considered statistically significant at *p* < 0.05. Levels of statistical significance were denoted as * *p* < 0.05, ** *p* < 0.01, *** *p* < 0.001, and **** *p* < 0.0001. Analyses and graph generation were performed using GraphPad Prism v.8.0.1 (San Diego, CA, USA).

## 5. Conclusions

This study provides an integrated evaluation of the antimicrobial, antivirulence, intracellular, immunomodulatory, and in silico predictive potential of DFO against Mabs. The findings demonstrate that DFO exerts predominantly bacteriostatic activity, reduces virulence-associated phenotypes, including biofilm formation and extracellular vesicle production, decreases the intracellular bacterial burden in RAW 264.7 macrophages, and modulates the expression of inflammatory mediators. Furthermore, its combination with antibiotics, particularly CLA, suggests a potential adjuvant effect associated with iron restriction and metabolic sensitization of the mycobacterium.

Taken together, these results indicate that DFO represents a promising adjuvant strategy based on the disruption of iron homeostasis in Mabs. However, interpretations should be approached with caution, as the in silico analyses are exploratory in nature and do not independently confirm direct inhibition of bacterial targets. Additional studies involving genetically diverse clinical isolates, iron-rescue assays, mature biofilm models, time-kill kinetics, human cell models, in vivo infection models, and biochemical validation of the targets predicted by molecular docking are required to confirm the proposed mechanism of action, strengthen the translational relevance of these findings, and establish the therapeutic applicability of DFO as an adjunctive treatment for Mabs infections.

## Figures and Tables

**Table 1:** Minimum Inhibitory Concentrations (MICs) and Minimum Bactericidal Concentrations (MBCs) of DFO, AMK, and CLA against Mabs.

Parameters	DFO	AMK	CLA
MIC	9.75 µg/mL	1 µg/mL	7 µg/mL
MBC	>10 mg/mL	1 µg/mL	7 µg/mL
MBC/MIC Ratio	>1.025	1	1
Activity	Bacteriostatic	Bactericidal	Bactericidal

DFO, deferoxamine; AMK, amikacin; CLA, clarithromycin; MIC, minimum inhibitory concentration; MBC, minimum bactericidal concentration. Values were obtained from three independent biological experiments, each performed in technical triplicate.

**Table 2:** In silico biological activity profiles of DFO, AMK, and CLA.

Activities	PASS Predictions	
**DFO**	**PA**	**PI**
Iron antagonist	0.911	0.000
CDP-glycerol glycerophosphotransferase inhibitor	0.875	0.014
Polyamine-transporting ATPase inhibitor	0.826	0.004
Yeast ribonuclease inhibitor	0.808	0.003
**AMK**		
CDP-glycerolglycerophosphotransferase inhibitor	0.955	0.003
Cellulose 1,4-beta-cellobiosidase inhibitor	0.943	0.000
Antiprotozoal (Leishmania)	0.883	0.003
Phosphoinositide 5-phosphatase inhibitor	0.859	0.003
Mucinaminylserine mucinaminidase inhibitor	0.846	0.004
Immunostimulant	0.843	0.007
Membrane integrity antagonist	0.831	0.005
Antibacterial	0.816	0.002
**CLA**		
CYP2H substrate	0.996	0.000
CYP3A1 substrate	0.993	0.000
CYP3A7 substrate	0.993	0.000
CYP3A2 substrate	0.991	0.000
CYP2B substrate	0.990	0.001
CYP2C11 substrate	0.987	0.001
Anti-infective	0.982	0.002

DFO: Deferoxamine; AMK: Amikacin; CLA: Clarithromycin; PA: probability of active effect; PI: probability of inactive effect; PASS: Prediction of Activity Spectra for Substances. Data were obtained through computational prediction via the PASS Online web server, based on molecular similarity models and machine learning algorithms.

**Table 3:** Estimated oral bioavailability, predicted toxic effects, absorption, and solubility of DFO compared with AMK and CLA.

Estimated Oral Bioavailability	DFO	AMK	CLA
LogP	4.33	1.84	2.44
MW (g/mol)	560.68	585.60	747.95
TPSA (Å^2^)	205.84	331.94	182.91
nHBD	6	13	4
nHBA	9	17	14
Molar refractivity	142.48	128.99	194.09
nRB	28	11	8
**Predicted toxic effects**			
Carcinogenicity	No	No	No
Cardiotoxicity	No	Yes	No
Cytotoxicity	No	No	No
Ecotoxicity	No	No	No
Hepatotoxicity	No	No	Yes
Immunotoxicity	No	Yes	Yes
Mutagenicity	Yes	No	No
Neurotoxicity	No	Yes	Yes
Nephrotoxicity	Yes	Yes	Yes
LD_50_ (mg/kg)	1.000	4.000	1.230
Ames toxicity	No	No	No
Acute oral toxicity	Low	Minimal	Low
Acute toxicity in rats	2.062	1.750	2.723
Toxicity class	4	5	4
**Estimated absorption**			
GI absorption	Low	Low	Low
BBB permeability	No	No	No
Log K_p_ (cm/s)	−11.22	−15.47	−8.62
**Predicted solubility**			
LogS	−0.14	2.23	−5.94

DFO: Deferoxamine; AMK: Amikacin; CLA: Clarithromycin; LogP: octanol–water partition coefficient; MW: molecular weight; TPSA: topological polar surface area; nHBD: number of hydrogen-bond donors; nHBA: number of hydrogen-bond acceptors; nRB: number of rotatable bonds; LD_50_: median lethal dose; GI: gastrointestinal; BBB: blood–brain barrier; Log K_p_: skin permeation coefficient; LogS: solubility. The data were obtained through computational prediction using the SwissADME server, based on molecular similarity models and machine learning algorithms.

**Table 4:** Molecular targets of Mabs used for docking with DFO.

Potential Target	Protein	Family	Mode of Action
Lipid metabolism	Enoyl-CoA hydratase/isomerase	MaoC-type hydratase	Impairment of β-oxidation of cholesterol-derived intermediates
Energy metabolism	Fumarase (fumarate hydratase)	Lyase	Disruption of the citric acid cycle through competition with the substrate (malate)
Intracellular survival/Resistance	Eis2 protein	N-acetyltransferase (GNAT family)	Inhibition of aminoglycoside acetylation and resistance mechanisms
Energy metabolism	F-ATP synthase ε subunit (Mabε)	ATP synthase (ε subunit)	Conformational inhibition of rotational coupling, affecting ATP synthesis
Energy metabolism	Fumarase (fumarate hydratase)	Lyase	Disruption of the citric acid cycle through competition with the substrate (malate)

## Data Availability

Dataset available on request from the authors.

## Supplementary Materials

The following supporting information can be downloaded at https://www.mdpi.com/article/10.3390/ijms27135789/s1.
