# Supplementary material for: Deferoxamine Exhibits Antimicrobial and Immunomodulatory Activity Against Mycobacterium abscessus: Integrated In Silico and In Vitro Evidence
Source: Int J Mol Sci. 2026 Jun 26;27(13):5789. doi: 10.3390/ijms27135789 (PMC13361824; doi:10.3390/ijms27135789)
Supplement: Supplementary file 1 [file ijms-27-05789-s001.zip › ijms-4297903-supplementary.pdf]

SUPPLEMENTARY MATERIAL

Deferoxamine Enhances Antimicrobial and Immunomodulatory Responses Against *Mycobacterium abscessus*: Integrated In Silico and In Vitro Evidence

Lira et al., 2025

SUPPLEMENTARY Table S1: Raw FICI matrices from the checkerboard of the experiments.

FICI Matrix - CLA + DFO							
**MIC DFO (µg/mL): 9.7500**							
**MIC CLA (µg/mL): 7.0000**							
	9.750	4.870	2.430	1.210	0.600	0.300	0.150
**FICI**	**DFO 9.75**	**DFO 4.87**	**DFO 2.43**	**DFO 1.21**	**DFO 0.6**	**DFO 0.3**	**DFO 0.15**
**CLA 7**	2.000	1.499	1.249	1.124	1.062	1.031	1.015
**CLA 3.5**	1.500	0.999	0.749	0.624	0.562	0.531	0.515
**CLA 1.75**	1.250	0.749	0.499	0.374	0.312	0.281	0.265
**CLA 0.875**	1.125	0.624	0.374	0.249	0.187	0.156	0.140
**CLA 0.4375**	1.063	0.562	0.312	0.187	0.124	0.093	0.078
**CLA 0.21875**	1.031	0.531	0.280	0.155	0.093	0.062	0.047

FICI Matrix - AMK + DFO							
**MIC DFO (µg/mL): 9.7500**							
**MIC CLA (µg/mL): 7.0000**							
	9.750	4.870	2.430	1.210	0.600	0.300	0.150
**FICI**	**DFO 9.75**	**DFO 4.87**	**DFO 2.43**	**DFO 1.21**	**DFO 0.6**	**DFO 0.3**	**DFO 0.15**
**AMK 1**	2.000	1.499	1.249	1.124	1.062	1.031	1.015
**AMK 0.5**	1.500	0.999	0.749	0.624	0.562	0.531	0.515
**AMK 0.25**	1.250	0.749	0.499	0.374	0.312	0.281	0.265
**AMK 0.125**	1.125	0.624	0.374	0.249	0.187	0.156	0.140
**AMK 0.0625**	1.063	0.562	0.312	0.187	0.124	0.093	0.078
**AMK 0.03125**	1.031	0.531	0.280	0.155	0.093	0.062	0.047

FICI Matrix - CLA + AMK							
**MIC AMK (µg/mL): 1.0000**							
**MIC CLA (µg/mL): 7.0000**							
	1.000	0.500	0.250	0.156	0.125	0.063	0.031
**FICI**	**AMK 1**	**AMK 0.5**	**AMK 0.25**	**AMK 0.15625**	**AMK 0.125**	**AMK 0.0625**	**AMK 0.03125**
**CLA 7**	2.000	1.500	1.250	1.156	1.125	1.063	1.031
**CLA 3.5**	1.500	1.000	0.750	0.656	0.625	0.563	0.531
**CLA 1.75**	1.250	0.750	0.500	0.406	0.375	0.313	0.281

**CLA 0.875**	1.125	0.625	0.375	0.281	0.250	0.188	0.156
**CLA 0.4375**	1.063	0.563	0.313	0.219	0.188	0.125	0.094
**CLA 0.21875**	1.031	0.531	0.281	0.188	0.156	0.094	0.063

Legend	Criteria
Synergistic	FICI ≤ 0.5
Additive	0.5 < FICI ≤ 1.0
Indifference	1.0 < FICI ≤ 4.0
Antagonism	FICI > 4.0

SUPPLEMENTARY Table S2: Gene Expression by qPCR

Primers Sequences Used

Gene	Direction	Sequence (5' → 3')	Length (bp)
TNF-α	Forward	GCCCAGGCAGTCAGATCATC	20
TNF-α	Reverse	CGCCACCATGAGCACTGAAAG	21
IL-1β	Forward	CTGAGGTGCTGATGTACCAG	20
IL-1β	Reverse	TAGATAGGCAGACAGCACGA	20
IL-6	Forward	TGACAAAGCCAGAGTCCTTC	20
IL-6	Reverse	CTACATTTGCCGAAGAGCCCC	21
IL-10	Forward	GGGAAGCTGAGAACCAAGAC	20
IL-10	Reverse	CAGTGAGGCGTACAGCCGTAG	21
IFN-γ	Forward	TGAATGACTGGAGTGCCAAG	20
IFN-γ	Reverse	CGACAGCAGCGACTCCTTTT	20
TGF-β	Forward	ACCCTGGATACCAACTATTG	20
TGF-β	Reverse	CGGGACTGATCCTAGTGTATT	21
β-actin	Forward	TGACAGGATGCAGAAGGAGA	20
β-actin	Reverse	TGCTGATCCACATCTGCTGGA	21

SUPPLEMENTARY REFERENCES

1. **Livak, K. J.; Schmittgen, T. D. Analysis of Relative Gene Expression Data Using Real-Time Quantitative PCR and the 2-ΔΔCT Method. methods 2001, 25 (4), 402–408.**
